# Impact of maternal lipopolysaccharide and polyinosinic-polycytidylic acid-induced infections on offspring cardiac development: Mitochondrial dysfunction and metabolic alterations

**DOI:** 10.1016/j.gendis.2025.101877

**Published:** 2025-10-08

**Authors:** Yingrui Li, Willfredius Mugishagwe Rutahoile, Binquan Xiong, Jianlin Du, Songbai Deng, Bin Liu, Xiaodong Jing, Huiping Yang, Yue Han, Qiang She

**Affiliations:** Department of Cardiology, The Second Affiliated Hospital of Chongqing Medical University, Chongqing 400010, China

**Keywords:** Cardiac development, Lipid peroxidation, Maternalinfection, Mitochondrial function, Oxidative stress

## Abstract

Maternal infections can have profound effects on embryonic heart development, yet the precise pathways through which these impacts manifest are still largely unexplored. This research explores the influence of maternal exposure to lipopolysaccharide (LPS) and polyinosinic-polycytidylic acid [Poly(I:C)] on metabolic profiles and mitochondrial function of offspring. At embryonic day 16.5, pregnant female C57BL/6J mice received either LPS or Poly(I:C) treatment. Human induced pluripotent stem cells were differentiated into cardiomyocytes (hiPSC-CMs) to evaluate the effects of various interventions on cardiomyocyte differentiation. mRNA sequencing and untargeted metabolomics were performed to analyze metabolic alterations. The findings from mRNA sequencing indicated that both LPS and Poly(I:C) caused metabolic pathway disturbances in the offspring's heart, with differentially expressed genes enriched in lipid, energy, and amino acid metabolism. Additionally, untargeted metabolomics showed a notable elevation in polyunsaturated fatty acids following LPS or Poly(I:C) treatment. Moreover, both LPS and Poly(I:C) treatment significantly impaired mitochondrial function, increased reactive oxygen species, and heightened lipid peroxidation within offspring mouse hearts. Mitochondrial dysfunction was mitigated through the application of antioxidant agents, such as N-acetylcysteine and ferrostatin-1. During hiPSC-CM differentiation, Poly(I:C) treatment led to similar mitochondrial dysfunction, while LPS treatment had minimal effects on ATP levels and lipid peroxidation. These findings indicate that maternal infection impairs metabolic signaling and mitochondrial function in the developing heart, with oxidative stress and lipid peroxidation playing key roles in these effects.

## Introduction

Cardiac development is a multi-step process that involves intricate biological mechanisms and strict developmental controls. During the fourth week of human cardiac development (approximately E10.5 in mice), the heart tube undergoes looping and forms the four-chambered structure. Subsequently, the embryonic heart continues to mature, and post-birth growth is driven by hypertrophy and cardiomyocyte proliferation,^1^^,^^2^ while metabolic pathway shifts also occur.^3^ The process of heart development encompasses more than just the structural changes; it also entails the differentiation and maturation of various cell types. Among these processes, cardiomyocyte differentiation stands out as vital, involving enhanced cardiac contractility, maturation of electrophysiological characteristics, changes in proliferation rates, and a shift in metabolic state from a reliance on glycolysis to an increased utilization of fatty acid metabolism.^4^^,^^5^ Recent studies underscore the essential impact of metabolic reprogramming on cardiomyocyte differentiation and maturation. A study indicates that certain proteins linked to metabolic pathways in the developing heart of fetal mice showed a marked increase during the later stages of heart development (E13.5–E16.5),^6^ suggesting that this developmental phase is key for metabolic reprogramming within fetal cardiac development. Inhibition of fatty acid oxidation in mice resulted in a significant alteration in the development trajectory of cardiomyocytes, with the emergence of immature cardiomyocytes, thereby suggesting that metabolic pathways may play a pivotal role in cardiomyocyte differentiation.^7^ Moreover, recent studies on human induced pluripotent stem cell-derived cardiomyocytes (hiPSC-CMs) suggest that various metabolic substances can significantly impact cardiomyocyte maturation, affecting mitochondrial biogenesis, transcriptional profiles, myofibril density and arrangement, and calcium handling.^8^, ^9^, ^10^ These studies highlight the profound impact of metabolic environment changes on cardiac development.

Maternal infection is strongly associated with embryonic heart development and can lead to a notable rise in the occurrence of congenital heart defects in offspring,^11^^,^^12^ whereas the molecular mechanisms are not yet fully understood. Lipopolysaccharide (LPS) and polyinosinic-polycytidylic acid [Poly(I:C)] are established Toll-like receptor (TLR) agonists (TLR4 and TLR3, respectively) widely employed to model gram-negative bacterial or viral maternal infections.^13^^,^^14^ LPS and Poly(I:C) interventions can lead to the production of pro- and anti-inflammatory cytokines, triggering a chain reaction that ultimately manifests as developmental defects in the offspring.^15^^,^^16^ Recent studies indicate that placental inflammation can result in structural anomalies in the developing fetal heart, including a reduction in left ventricular myocardial thickness and a diminished rate of cardiomyocyte proliferation,^17^ underscoring the critical role of infection in modulating cardiac growth. Previous research has also shown that immune activation triggered by diverse factors can significantly influence cardiac metabolism. For example, LPS or certain infections can stimulate macrophages to generate itaconate, which facilitates metabolic reprogramming through direct modifications of cysteine residues within glycolytic enzymes, thereby impairing function of glycolysis.^18^ Activation of TLRs can trigger the production of phosphatidylcholine within macrophages, which in turn enhances the inflammatory response.^19^ However, whether maternal infection affects the metabolic environment during offspring heart development has not been reported.

Mitochondria are fundamental to the cellular metabolic system, participating in various metabolic processes, including glucose metabolism and lipid metabolism. Cardiomyocyte differentiation involves up-regulation of the gene peroxisome proliferator-activated receptor gamma coactivator 1-alpha (Ppargc1a), a critical regulator of mitochondrial biogenesis, which drives cardiomyocyte maturation.^20^ Conversely, reducing the mRNA expression of the mitochondrial fission protein dynamin-related protein 1 (Drp1) significantly hampers the capacity of stem cells to differentiate into cardiomyocytes.^21^ Meanwhile, reactive oxygen species (ROS), generated as by-products of mitochondrial metabolism, appear to be crucial in cardiomyocyte differentiation. In general, ROS are produced by multiple cellular organelles, including mitochondria, the endoplasmic reticulum, and the plasma membrane. During infection and inflammation, ROS levels increase significantly, ultimately resulting in irreversible cellular damage. During cardiomyocyte differentiation, elevated levels of ROS can inhibit the self-renewal capacity of stem cells and promote their differentiation into cardiomyocytes, while antioxidants have been shown to decrease ROS and support cardiomyocyte differentiation at E9.5 during development.^22^ This implies that maintaining an optimal level of ROS is essential for successful differentiation of cardiomyocytes. Within a specific concentration range, ROS production supports the cardiac fate of pluripotent stem cells. However, once the concentration of ROS exceeds a certain threshold, it initiates a detrimental cascade that ultimately impairs the differentiation process.

The objective of this research is to simulate maternal infections caused by different pathogens using LPS and Poly(I:C), and to investigate their impact on metabolic alterations and mitochondrial function during heart development in offspring mice, as well as the role of oxidative stress in these processes.

## Materials and methods

All animal experiments were adhered strictly to the ARRIVE guidelines and regulations on laboratory animal management by the National Research Council's Guide for the Care and Use of Laboratory Animals, and were approved by the Institutional Animal Care and Use Committee (IACUC) of the Second Affiliated Hospital of Chongqing Medical University (IACUC-SAHCQMU-2024-00013).

A detailed description of the methods used in this study is provided in the supplementary materials. In brief, pregnant mice were administered LPS and Poly(I:C) injections at E16.5. The hearts of newborn offspring mice (within 12 h of birth, postnatal day [P]0) were collected for further analysis. hiPSC lines, purchased from Beijing Cellapy Biotechnology (China), were differentiated into hiPSC-CMs. LPS or Poly(I:C) was applied during hiPSC-CM differentiation (days 16–20), and cells at day 20 were used for subsequent experiments to observe the effects on cardiomyocyte differentiation. The mRNA sequencing and nontargeted metabolomics were performed, with the detailed datasets presented in Supplemental Tables S1 and S2, respectively. The primer sequences used for qPCR are listed in Supplemental Table S3. An unpaired Student's *t*-test was used for comparisons between two independent groups. For comparisons involving more than two groups, a one-way analysis of variance (ANOVA) followed by a Holm-Sidak post-test for multiple comparisons was conducted. *P*-values < 0.05 were considered statistically significant.

## Results

### Maternal infection significantly impacts the cardiac metabolism of offspring mice

**Figure 1:**
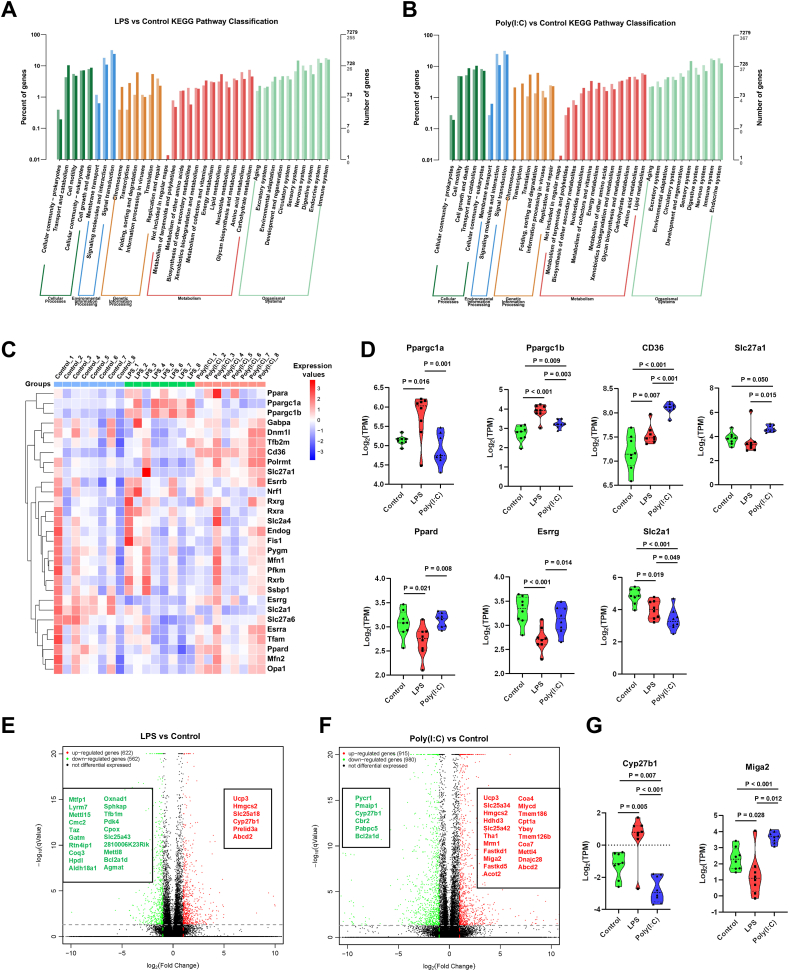
Maternal infections significantly affect cardiac metabolism in offspring mice. Pregnant mice were intraperitoneally injected with LPS (300 μg/kg) or Poly(I:C) (20 mg/kg) at E16.5. The hearts of the newborn offspring mice were extracted within 12 h of birth [postnatal day (P)0] for mRNA sequencing and bioinformatic analysis. **(A, B)** KEGG pathway classification histograms of differentially expressed genes in LPS versus control and Poly(I:C) versus control. The horizontal axis represents functional categories, while the vertical axis shows the number of genes within each category, with light colors representing differential genes and dark colors representing all genes. The left vertical axis shows the proportion of genes annotated to that function (differential genes/all genes). **(C)** Heatmap of metabolism-related genes among control, LPS, and Poly(I:C). **(D)** Violin plots of expression of metabolism-related genes among control, LPS, and Poly(I:C). **(E)** Volcano plot of differentially expressed genes in LPS versus control. **(F)** Volcano plot of differentially expressed genes in Poly(I:C) versus control. **(G)** Violin plots of expression of mitochondrial-related genes among control, LPS, and Poly(I:C). One-way analysis of variance (ANOVA) followed by Holm-Sidak post-test for multiple comparisons was used for comparing more than two groups. The data were presented as mean ± standard error.

To explore the influence of maternal infection on cardiac development in offspring mice, we treated pregnant mice at gestational day E16.5 with either LPS or Poly(I:C). We obtained neonatal offspring heart tissue for transcriptome sequencing analysis. Global transcriptomic profiling identified substantial gene expression perturbations in neonatal hearts upon maternal LPS or Poly(I:C) exposure (LPS: 622 up, 562 down; Poly(I:C): 915 up, 980 down). Gene ontology analysis highlighted significant involvement in developmental processes, particularly in terms related to multicellular organism development, developmental processes, and system development (Fig. S1A and S1B). Kyoto Encyclopedia of Genes and Genomes (KEGG) pathway classification analysis further pinpointed prominent dysregulation in core metabolic pathways, including lipid, energy, and amino acid metabolism in both groups (Fig. 1A and B). Additionally, KEGG enrichment highlighted specific metabolic pathways such as glycolysis/gluconeogenesis and arginine and proline metabolism in the LPS versus control group, while the PPAR signaling pathway and fatty acid elongation were enriched in the Poly(I:C) versus control group (Fig. S1C and S1D). These findings suggest that distinct maternal infections significantly impact offspring mice's cardiac metabolism.

We further screened the literature to identify several key genes involved in metabolic processes and analyzed the influence of maternal infection on the transcriptional expression of these genes (Fig. 1C). Interestingly, multiple genes related to lipid metabolism, including Ppargc1a, peroxisome proliferator-activated receptor gamma coactivator 1-beta (Ppargc1b), cluster of differentiation 36 (CD36), solute carrier family 27 member 1 (Slc27a1), and peroxisome proliferator-activated receptor delta (Ppard), showed significant changes. Other notable changes included the estrogen-related gene estrogen-related receptor gamma (Esrrg) and the glucose metabolism-related gene solute carrier family 2 member 1 (Slc2a1) (Fig. 1D).

Since mitochondria play a central role in cellular metabolic networks, we also explored the expression changes of mitochondria-related genes under different maternal infections. A list of relevant mitochondrial genes in mice was obtained from MitoCarta3.0. Mitochondrial gene expression diverged significantly: LPS exposure induced up-regulation of 6 and down-regulation of 20 genes, whereas Poly(I:C) triggered 21 up-regulated and 6 down-regulated genes relative to controls (Fig. 1E and F). These results indicate that while different maternal infections significantly alter the expression of mitochondrial-related genes, their effects vary, even showing opposite patterns for some genes. For instance, cytochrome P450 family 27 subfamily B member 1 (Cyp27b1) expression was significantly increased in the LPS versus control group but decreased in the Poly(I:C) versus control group, whereas mitoguardin 2 (Miga2) was significantly reduced in the LPS versus control group but increased in the Poly(I) versus control group (Fig. 1G).

### Maternal infection significantly inhibits cardiomyocyte proliferation in offspring hearts

The maturation of cardiomyocyte sarcomeres and the shift in their proliferative state are equally important for cardiac development in offspring. We next examined the effects of different maternal infections on sarcomere structure and proliferation in neonatal mice's hearts. Transcriptome sequencing analysis revealed altered expression of certain sarcomere-related genes in the Poly(I:C) versus control group, including significant reductions in myosin light chain 7 (Myl7) and titin (Ttn) and increases in actin alpha 1 (Acta1) and myozenin 2 (Myoz2) (Fig. S2A and S2B). However, key sarcomere-related genes, such as troponin T type 2 (Tnnt2), troponin I type 3 (Tnni3), and myosin heavy chain 6 (Myh6), did not show significant changes in either the LPS versus control group or Poly(I:C) versus control group, a finding further validated by quantitative reverse transcription PCR and Western blot analyses (Fig. S2A–S2E). Sarcomere length, important for mature cardiac contraction, was also comparable between infection and control groups (Fig. S2F and S2G). These results suggest that maternal infection does not significantly affect sarcomere maturation in offspring mice's hearts.

We further investigated the impact of maternal infection on cardiac proliferation in neonatal offspring. Transcriptome sequencing analysis showed a significant reduction in proliferation-related genes in both the LPS versus control group and Poly(I:C) versus control group, including marker of proliferation Ki-67 (Mki67), cyclin E1 (Ccne1), E2F transcription factor 1 (E2f1), and cyclin-dependent kinase 2 (Cdk2) (Fig. S3A and S3B). Immunofluorescence analysis confirmed a significant decrease in KI67 expression in neonatal cardiomyocytes from infection groups compared with the control, indicating a marked suppression of cardiac proliferation in the infection groups (Fig. S3C and S3D). Since cardiomyocyte proliferation remains critical for heart development after birth, we further assessed heart size in offspring mice at four weeks of age. In the LPS versus control group, offspring showed significant reductions in both body and heart weights, while in the Poly(I:C) versus control group, heart weight was significantly reduced (Fig. S3E and S3F). These findings suggest that maternal infection suppresses cardiac proliferation in offspring mice, potentially affecting heart growth during later development.

### Maternal infection impairs offspring mice's cardiac mitochondrial function

**Figure 2:**
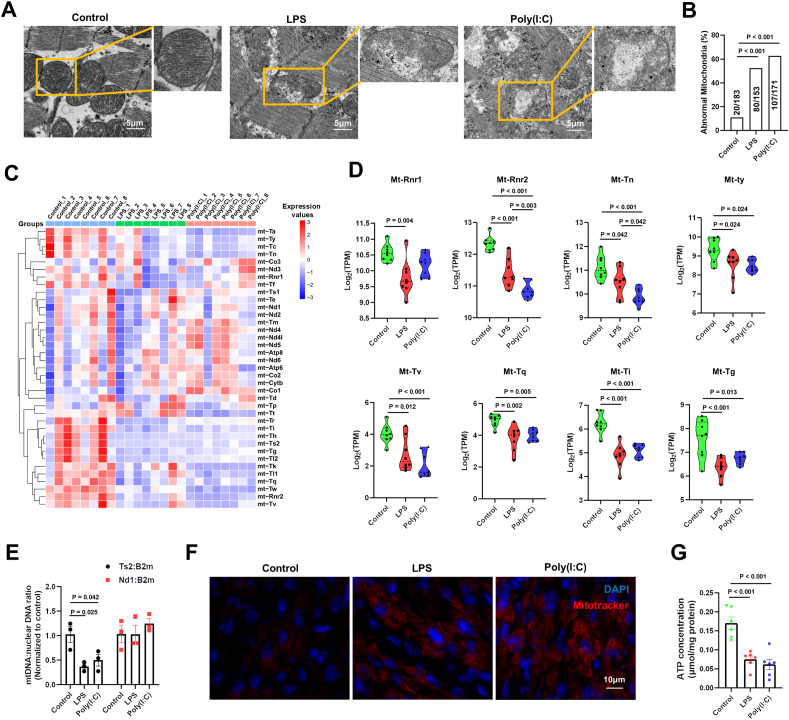
Maternal infections significantly affect mitochondrial function in offspring mouse hearts. Pregnant mice were intraperitoneally injected with LPS (300 μg/kg) or Poly(I:C) (20 mg/kg) at E16.5. The hearts of the newborn offspring mice were extracted within 12 h of birth [postnatal day (P)0] for mRNA sequencing and bioinformatic analysis. **(A)** Representative images of mitochondria in heart tissue of offspring mice (bar = 5 μm). **(B)** Quantification of abnormal mitochondria in the control, LPS, and Poly(I:C) groups. **(C)** Heatmap of mtDNA-related genes among the control, LPS, and Poly(I:C) groups. **(D)** Violin plots of expression of mtDNA-related genes among the control, LPS, and Poly(I:C) groups. **(E)** Mitochondrial Nd1 to nuclear B2M ratio and Ts2 to nuclear B2M DNA ratio in heart tissue of the control, LPS, and Poly(I:C) groups. **(F)** Representative images of heart tissue stained with Mitotracker/DAPI (bar = 10 μm). **(G)** ATP content in the control, LPS, and Poly(I:C) groups. One-way analysis of variance (ANOVA) followed by Holm-Sidak post-test for multiple comparisons was used for comparing more than two groups. The Fisher's test was applied for comparing categorical variables. The data were presented as mean ± standard error.

Mitochondria play a central role in cardiac metabolism, so we further investigated the effects of LPS and Poly(I:C) on mitochondrial function. Transmission electron microscopy revealed that, compared with the control group, maternal infection with either LPS or Poly(I:C) resulted in mitochondrial morphological changes, including swelling, cristae disorganization, and overall mitochondrial damage (Fig. 2A and B). Transcriptome analysis also showed that both LPS and Poly(I:C) led to a significant reduction in the expression of several mitochondrial transfer RNAs (mt-tRNAs) and mitochondrial ribosomal RNAs (mt-rRNAs), while the expression of mitochondrial messenger RNAs (mt-mRNAs) remained unaffected (Fig. 2C and D). Furthermore, we observed a decreased mitochondrial mt-Ts2 to nuclear DNA ratio, but no change in the mitochondrial Nd1 to nuclear DNA ratio, consistent with the transcriptome analysis results (Fig. 2E). Using Mito-Tracker Red CMXRos to label active mitochondria, we found that maternal infections caused an abnormal increase in mitochondrial membrane potential in cardiomyocytes, suggesting membrane instability and potential mitochondrial damage (Fig. 2F). As expected, since mitochondria are key organelles for ATP production, we observed lower ATP levels in both the LPS versus control group and Poly(I:C) versus control group, indicating that maternal infection impaired mitochondrial function in the offspring heart (Fig. 2G).

### Oxidative stress plays an important role in the effects of maternal infection on mitochondrial function

**Figure 3:**
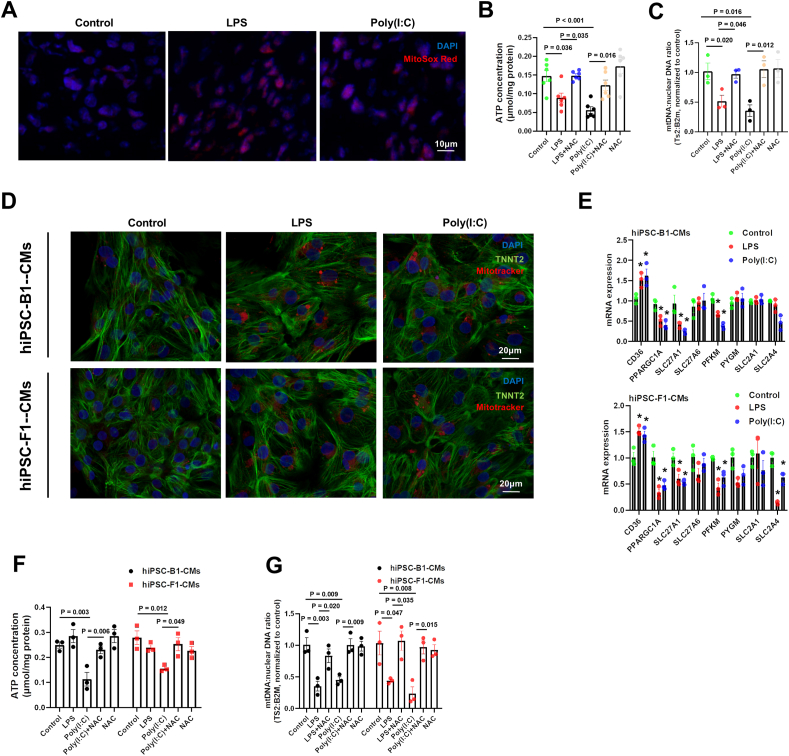
NAC counteracts the effects of LPS and Poly(I:C) on the hearts of offspring mice and hiPSC-CM differentiation. Pregnant mice were intraperitoneally injected with LPS (300 μg/kg), Poly(I:C) (20 mg/kg), or NAC (300 mg/kg) at E16.5. The hearts of the newborn offspring mice were extracted within 12 h of birth [postnatal day (P)0] for mRNA sequencing and bioinformatic analysis. For hiPSC differentiation into cardiomyocytes, LPS (5 μg/mL), Poly(I:C) (10 μg/mL), or NAC (10 μM) was applied from day 16 to day 20 of differentiation. On day 20 of differentiation, cells were used for experiments. **(A)** Representative images of heart tissue stained with MitoSox Red/DAPI in each group (bar = 10 μm). **(B)** ATP content in offspring mouse heart tissue in each group. **(C)** The Ts2 to nuclear B2M DNA ratio in offspring mouse heart tissue in each group. **(D)** Representative images of hiPSC-B1-CM and hiPSC-F1-CM cell lines stained with Mitotracker/TNNT2/DAPI (bar = 20 μm). **(E)** The fold change of mRNA expression levels of metabolic pathway-related genes in hiPSC-B1-CM and hiPSC-F1-CM cell lines with or without LPS or Poly(I:C) challenge. **(F)** ATP content in hiPSC-B1-CM and hiPSC-F1-CM cell lines in each group. **(G)** Ts2 to nuclear B2M DNA ratio in hiPSC-B1-CM and hiPSC-F1-CM cell lines in each group. One-way analysis of variance (ANOVA) followed by Holm-Sidak post-test for multiple comparisons was used for comparing more than two groups (∗*P* < 0.05). The data were presented as mean ± standard error.

Infection and inflammation significantly increase oxidative stress levels, which are closely related to mitochondrial function. To further investigate whether maternal infection affects mitochondrial function in offspring mice's hearts through abnormal oxidative stress, we used MitoSox Red to measure mitochondrial ROS levels in the hearts of neonatal offspring mice. Both the LPS versus control group and the Poly(I:C) versus control group exhibited elevated mitochondrial ROS levels (Fig. 3A). We then applied the antioxidant N-acetylcysteine (NAC) to counteract oxidative stress. NAC rescued the mitochondrial damage caused by LPS and Poly(I:C), including reduced ATP levels and a decreased mitochondrial mt-Ts2 to nuclear DNA ratio (Fig. 3B and C). To further observe the effects of LPS and Poly(I:C) on cardiomyocyte differentiation, we used two healthy hiPSC cell lines (B1 and F1) with obvious pluripotent markers nanog homeobox 1 (NANOG1) and octamer-binding transcription factor 4 (OCT4) (Fig. S4A). These cells were induced to differentiate into cardiomyocytes *in vitro* using a specific differentiation protocol. Both induced cardiomyocyte lines showed expression of the cardiac marker TNNT2 at 20 days of differentiation (Fig. S4B), along with an increase in the percentage of TNNT2-positive cells (Fig. S4C and S4D). The expression of other cardiomyocyte markers in both B1 and F1 lines did not show significant differences (Fig. S4E), suggesting that hiPSCs could effectively differentiate into cardiomyocytes and that there were no major differences between the two cell lines.

We found that LPS and Poly(I:C) also caused abnormal increases in membrane potential during cardiomyocyte differentiation *in vitro* (Fig. 3D). Additionally, quantitative reverse transcription PCR analysis showed that LPS and Poly(I:C) led to abnormal expression changes in metabolic and proliferation-related molecules in differentiated cardiomyocytes (Fig. 3E; Fig. S5A), including CD36, PPARGC1A, SLC27A1, phosphofructokinase (PFKM), MKI67, and E2F1. However, these expression changes varied between the different cell lines. For example, cyclin A1 (Ccna1) showed a decrease only in the F1 line, not in B1, which could be due to the differences between the healthy individuals from whom the cell lines were derived. We also observed that LPS and Poly(I:C) caused an increase in MitoSox Red levels in both B1 and F1, indicating that both infections led to increased oxidative stress levels in the differentiated cardiomyocytes (Fig. S5B). Poly(I:C) caused a reduction in ATP levels and mitochondrial MT-TS2 to nuclear DNA ratio in both cell lines, and NAC could rescue these phenotypes (Fig. 3F and G). Unexpectedly, LPS treatment did not cause significant changes in ATP levels, but it did result in a decreased mitochondrial MT-TS2 to nuclear DNA ratio, which was also rescued by NAC (Fig. 3F and G). Collectively, oxidative stress mediates maternal infection-induced mitochondrial dysfunction in the fetal heart. Distinct pathogen-specific mechanisms drive these perturbations, as evidenced by differential responses to bacterial (LPS) versus viral (Poly(I:C)) mimetics.

### Maternal infection leads to abnormal glycerophospholipid metabolism and promotes lipid peroxidation

**Figure 4:**
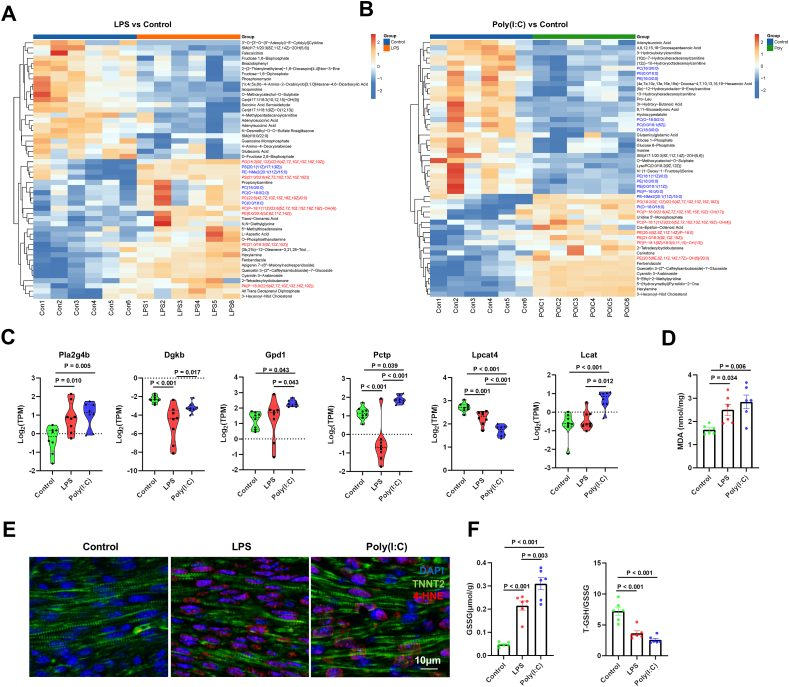
Maternal infection increases lipid peroxidation levels in offspring mouse hearts. Pregnant mice were intraperitoneally injected with LPS (300 μg/kg) or Poly(I:C) (20 mg/kg) at E16.5. The hearts of the newborn offspring mice were extracted within 12 h of birth [postnatal day (P)0] for mRNA sequencing and bioinformatic analysis. **(A)** Heat map of differential metabolites in LPS versus control. **(B)** Heat map of differential metabolites in LPS versus Poly(I:C). **(C)** Violin plots of the expression of phospholipid metabolism-related genes among control, LPS, and Poly(I:C). **(D)** Malonaldehyde (MDA) content in offspring mouse heart tissue in each group. **(E)** Representative images of heart tissue stained with 4-HNE/TNNT2/DAPI in each group (bar = 10 μm). **(F)** Glutathione disulfide (GSSG) and total glutathione (T-GSH)/GSSG in offspring mouse heart tissue in each group. One-way analysis of variance (ANOVA) followed by Holm-Sidak post-test was used for comparing more than two groups. The data were presented as mean ± standard error.

Given that maternal infection significantly affects metabolic pathways in the hearts of offspring mice, we further conducted gas chromatography-mass spectrometry and liquid chromatography-mass spectrometry nontargeted metabolomics analysis on the hearts of neonatal offspring. In the LPS versus control group, we identified 89 up-regulated metabolites and 53 down-regulated metabolites; in the Poly(I:C) versus control group, we identified 88 up-regulated metabolites and 159 down-regulated metabolites (Fig. S6A and S6B). KEGG analysis of these differential metabolites revealed that both the LPS and Poly(I:C) groups were enriched in pathways such as ABC transporters, glycerophospholipid metabolism, and oxidative phosphorylation (Fig. S6C and S6D). Interestingly, we found that both groups showed significant increases in various glycerophospholipids containing polyunsaturated fatty acids, such as phosphatidylglycerols (18:2(9Z,12Z)/22:6(4Z,7Z,10Z,13Z,16Z,19Z)) and phosphatidylethanolamines (21:0/18:3(9Z,12Z,15Z)), which were significantly elevated in both groups (Fig. 4A and B). Additionally, in the Poly(I:C) group, several glycerophospholipids, including both saturated and monounsaturated fatty acids, showed significant decreases (Fig. 4B). We further identified genes associated with glycerophospholipid metabolism in the GSEA-Molecular Signatures Database and observed changes in these molecules in the two groups through bioinformatic analysis of mRNA sequencing. Multiple glycerophospholipid metabolism-related molecules were significantly altered, indicating a potential impact of different maternal infections on glycerophospholipid metabolism (Fig. 4C).

Glycerophospholipids are key components of cell membranes, and the increase of polyunsaturated fatty acids in cell membranes, along with elevated ROS, is an important inducer of lipid peroxidation. Therefore, we further investigated whether maternal infections led to lipid peroxidation in the hearts of offspring mice. We found that, in both LPS and Poly(I:C) groups, lipid peroxidation indicators, such as malonaldehyde (MDA) and 4-hydroxynonenal (4-HNE), were significantly elevated (Fig. 4D and E). Meanwhile, glutathione disulfide (GSSG) was also significantly increased in both groups, and the total glutathione (T-GSH)/GSSG ratio was reduced (Fig. 4F). These results suggest that both LPS and Poly(I:C) induce lipid peroxidation.

### Maternal infection induces lipid peroxidation-mediated mitochondrial damage in offspring hearts

**Figure 5:**
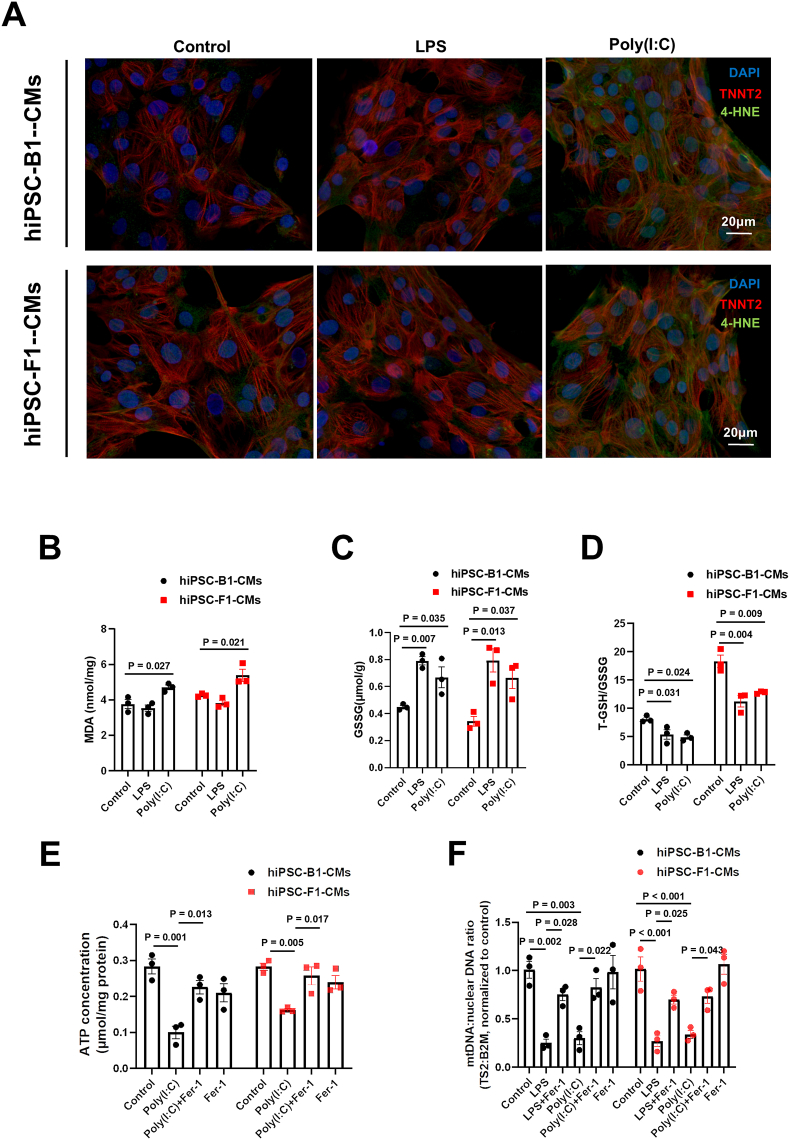
Poly(I:C) challenge, but not LPS, impairs mitochondrial function in the cardiac differentiation process by increasing lipid peroxidation levels. From day 16 to day 20 of hiPSC differentiation into cardiomyocytes, LPS (5 μg/mL), Poly(I:C) (10 μg/mL), and Fer-1 (2 μM) treatments were applied. On day 20 of differentiation, cells were used for the experiments. **(A)** Representative images of hiPSC-B1-CM and hiPSC-F1-CM cell lines stained with 4-HNE/TNNT2/DAPI (bar = 20 μm). **(B)** Malonaldehyde (MDA) content in hiPSC-B1-CM and hiPSC-F1-CM cell lines with or without LPS or Poly(I:C) challenge. **(C)** Glutathione disulfide (GSSG) in hiPSC-B1-CM and hiPSC-F1-CM cell lines with or without LPS or Poly(I:C) challenge. **(D)** Total glutathione (T-GSH)/GSSG in hiPSC-B1-CM and hiPSC-F1-CM cell lines with or without LPS or Poly(I:C) challenge. **(E)** ATP content in hiPSC-B1-CM and hiPSC-F1-CM cell lines in each group. **(F)** Ts2 to nuclear B2M DNA ratio in hiPSC-B1-CM and hiPSC-F1-CM cell lines in each group. One-way analysis of variance (ANOVA) followed by Holm-Sidak post-test for multiple comparisons was used for comparing more than two groups. The data were presented as mean ± standard error.

To further investigate whether lipid peroxidation induced by maternal infection mediates mitochondrial damage in the hearts of offspring mice, we used ferrostatin-1 (Fer-1), a phenolic antioxidant compound, which was found to effectively prevent lipid peroxidation and ferroptosis. We found that Fer-1 exhibited similar effects to NAC, rescuing mitochondrial damage caused by LPS and Poly(I:C), including restored ATP levels and mitochondrial mt-Ts2 to nuclear DNA ratio (Fig. S6E and S6F), highlighting the important role of lipid peroxidation in this process. Furthermore, using the B1 and F1 cell lines, we examined whether LPS and Poly(I:C) could also induce lipid peroxidation during the differentiation of hiPSCs into cardiomyocytes *in vitro*. Interestingly, we only observed abnormal increases in lipid peroxidation markers 4-HNE and MDA after Poly(I:C) treatment (Fig. 5A and B). Additionally, GSSG was significantly elevated in both groups, and the T-GSH/GSSG ratio was significantly reduced, likely due to increased ROS generation and oxidative stress (Fig. 5C and D). Importantly, Fer-1 treatment also rescued mitochondrial damage caused by Poly(I:C), including restored ATP levels and mitochondrial MT-TS2 to nuclear DNA ratio (Fig. 5E and F).

## Discussion

Our findings indicate that maternal infection induced by LPS or Poly(I:C) challenge significantly influences fetal cardiac development, including inhibition of myocardial proliferation and disruption of mitochondrial function, where oxidative stress and lipid peroxidation act as pivotal contributors.

The results indicate that maternal infection caused by LPS and Poly(I:C) exposure leads to significant alterations in the cardiac metabolic pathways and mitochondrial function within neonatal offspring mice. Moreover, it exerts an inhibitory effect on myocardial proliferation. Specifically, both types of maternal infection led to disrupted mitochondrial morphology, abnormal membrane potential, and diminished ATP production in the offspring mice's hearts. Furthermore, both LPS and Poly(I:C) administration stimulated the production of cardiac mitochondrial ROS and elevated markers of lipid peroxidation, such as elevated levels of polyunsaturated fatty acids, MDA, and 4-HNE. Notably, treatment with the antioxidant NAC, as well as Fer-1, a lipid peroxidation inhibitor, mitigated mitochondrial damage in offspring mice, suggesting that lipid peroxidation may mediate mitochondrial impairment in response to maternal infection. During hiPSC-CM differentiation, although both LPS and Poly(I:C) challenges led to a reduced mitochondrial mt-Ts2 to nuclear DNA ratio along with increased levels of mitochondrial ROS, only the Poly(I:C) challenge reduced ATP levels. In contrast, the LPS challenge did not decrease ATP levels or elevate concentrations of 4-HNE and MDA. These *in vitro* findings suggest that lipid peroxidation may influence ATP production during cardiomyocyte differentiation.

The development of the heart is a multifaceted, sequential process that is significantly influenced by metabolic changes. During the initial stages of heart development, glucose and lactate are the primary substrates that provide ATP, while both contribute significantly to cell proliferation. During myocardial maturation, glycolysis decreases while β-oxidation of fatty acids increases simultaneously.^23^ Recent research on the embryonic mouse proteome revealed that proteins involved in metabolic pathways, such as glycolysis, fatty acid oxidation, and oxidative phosphorylation, are notably elevated in late-stage embryonic heart development.^6^ Our transcriptomic analysis reveals that exposure to LPS or Poly(I:C) leads to profound changes in the cardiac metabolic pathways in offspring mice, including pathways and molecules related to lipid and glucose metabolism. Recent studies suggest that alterations in key metabolic enzymes, such as fatty acid transporter carnitine palmitoyltransferase 1b (Cpt1b) or Ppard, or transient lactate exposure, can notably impact cardiomyocyte proliferation and differentiation.^7^^,^^24^ Interestingly, both LPS and Poly(I:C) groups led to a significant reduction of KI67 expression, suggesting that these stimuli suppressed cell proliferation. This result aligns with a previous study that indicates reduced myocardial thickness and diminished myocardial cell proliferation,^17^ potentially due to the influence of maternal infection on glucose and lipid metabolism. Previous studies have indicated that metabolic reprogramming significantly impacts cardiomyocyte proliferation. For example, the inhibition of fatty acid oxidation in cardiomyocytes stimulates cardiomyocyte proliferation.^7^ Another study showed that inactivation of Drp1 in cardiomyocytes during embryonic development resulted in mitochondrial abnormalities and decreased cell proliferation.^25^ Furthermore, in the adult zebrafish heart model, stimulation of glycolysis in heart tissues adjacent to the damaged area promotes cardiomyocyte proliferation.^26^ These findings are consistent with our own results, suggesting that pregnancy-related infection may inhibit cardiomyocyte proliferation in the offspring by affecting metabolic pathways.

Both LPS and Poly(I:C) similarly led to significant alterations in specific mitochondrial-related genes. For instance, the mitochondria-regulatory protein uncoupling protein-3 (UCP3), known for its ability to facilitate proton transport and uncoupling activity, showed abnormal elevation in both groups. The specific physiological function of UCP3 within the cardiac energy metabolism remains largely unclear, though it is known to facilitate fatty acid transport and oxidation within mitochondria while decreasing mitochondrial ROS production.^27^ Interestingly, our findings display increased levels of mitochondrial ROS in both the LPS and Poly(I:C) groups, suggesting that the observed rise in UCP3 expression might represent a feedback response to elevated ROS. Miga2, which encodes a mitochondrial outer membrane protein, plays an essential role in lipid transport, especially in transporting phospholipids between membranes.^28^ However, Miga2 exhibited differential expression in the Poly(I:C) and LPS groups: it increased following Poly(I:C) treatment but decreased after LPS treatment. Under Poly(I:C)-induced pathological stress, Miga2 overexpression might facilitate enhanced or abnormal lipid metabolism in response to heightened energy demands or cellular stress, potentially affecting cell function. Conversely, LPS may reduce Miga2 expression, possibly impairing lipid transport and mitochondrial function due to cell damage. However, the precise mechanisms underlying these effects require further investigation. These findings suggest that both LPS- and Poly(I:C)-induced maternal infections significantly impact metabolism and mitochondrial function-related molecules in the offspring's heart, thereby influencing cardiac growth.

Mitochondria, known as the cell's “powerhouse”, generate substantial amounts of ATP through oxidative phosphorylation and mediate various cellular metabolic processes. During cardiomyocyte differentiation, the primary energy source gradually shifts from high glycolysis dependence to increased mitochondrial oxidative phosphorylation, mainly involving fatty acid oxidation and minimal glucose oxidation, to meet ATP demands, highlighting mitochondria's essential role in cardiac maturation.^29^ Multiple factors influence mitochondrial function, including proper mtDNA coding, mitochondrial membrane stability, and the accumulation of mitochondrial ROS. Mouse mtDNA, which is a compact double-stranded circular molecule, contains 37 distinct genes. Among these, there are 22 transfer RNAs (tRNAs), 2 ribosomal RNAs (rRNAs), and 13 polypeptides. The 13 polypeptides constitute the protein subunits of oxidative phosphorylation complexes I, III, IV, and V, while tRNAs and rRNAs are essential for translating these mtDNA-encoded polypeptides.^30^ As adapters decoding mRNA into proteins, mt-tRNAs are crucial for maintaining mitochondrial function; defects in mt-tRNA can lead to respiratory deficiencies and various mitochondrial diseases.^31^ Our findings reveal that maternal infection challenges significantly reduce multiple mt-rRNA and mt-tRNA expression, while the 13 mitochondrial respiratory chain peptides remain unchanged. This suggests that maternal infection may primarily impact mt-tRNA and mt-rRNA translation processes. These alterations may be driven by increased mitochondrial ROS due to infection challenges, as our animal and cell models exhibit decreased mitochondrial mt-Ts2 to nuclear DNA ratios alongside elevated mitochondrial ROS, both of which can be rescued by the antioxidant NAC. This aligns with previous research indicating that mtDNA copy numbers are vulnerable to oxidative damage from mitochondrial ROS.^32^ However, the specific role of each mt-tRNA in the translation process remains unclear, and subsequent study is needed to determine the precise impacts of decreased mt-tRNA on mitochondrial function following LPS and Poly(I:C) challenges.

The mitochondrial membrane potential (ΔΨm) originates from redox transformations linked to the Krebs cycle activity, closely associated with ATP production. These transformations generate electrical potential and a proton gradient, collectively forming the transmembrane potential of hydrogen ions.^33^ ΔΨm stability is essential for maintaining normal cellular functions. Prolonged, inappropriate low ΔΨm may result in inadequate ATP production and reductive stress from low mitochondrial ROS levels. Conversely, an excessively high ΔΨm leads to significant ROS production within the mitochondrial respiratory chain, which can cause further mitochondrial damage.^34^^,^^35^ Our findings indicate that both LPS and Poly(I:C) challenges lead to abnormally elevated ΔΨm and mitochondrial ROS in the hearts of offspring mice and in differentiating hiPSC-CMs *in vitro*. This elevated mitochondrial ROS may partially originate from the increased ΔΨm. Treatment with NAC effectively rescues mitochondrial damage in offspring mouse hearts and hiPSC-CMs caused by LPS and Poly(I:C) challenges, including ATP reduction and the decreased mitochondrial mt-Ts2 to nuclear DNA ratio, suggesting that elevated ROS further exacerbates mitochondrial damage, consistent with previous findings.^36^^,^^37^

Interestingly, in the *in vitro* model, LPS challenge did not significantly reduce ATP levels during cardiomyocyte differentiation. A likely reason is that the observed reduction in ATP levels triggered by LPS or Poly(I:C) during cardiomyocyte differentiation depends on lipid peroxidation. Supporting evidence is that although *in vitro* LPS challenge induces mitochondrial ROS production, it does not trigger lipid peroxidation, whereas both *in vitro* Poly(I:C) challenge and *in vivo* offspring mouse heart models do induce lipid peroxidation, leading to significant ATP reduction. This difference between *in vivo* and *in vitro* findings may stem from the distinct immune environments generated by LPS and Poly(I:C) when administered in these two distinct experimental settings. *In vivo*, LPS and Poly(I:C) treatments trigger strong immune responses, activating the immune system and encouraging the generation of pro-inflammatory factors, such as interleukins (like IL-1, IL-6) and tumor necrosis factors (like TNF-α).^14^^,^^38^ Although previous studies suggest that LPS and Poly(I:C) challenges can stimulate inflammation to some extent *in vitro*,^39^^,^^40^ there are still notable differences from the immune activation environment *in vivo*. This discrepancy might explain why ATP reduction and lipid peroxidation were not induced in response to *in vitro* LPS treatment. Another possible explanation for the observed differences is the disparity between *in vitro* hiPSC-CM differentiation and *in vivo* cardiomyocyte maturation. The immature phenotype of hiPSC-CMs leads to different expression profiles of ion channels, sarcomeric proteins, and metabolic enzymes.^41^ This could mean that the antioxidant mechanisms in hiPSC-CMs may not yet be fully developed or sufficiently adapted to manage oxidative stress induced by LPS, which could explain the lack of lipid peroxidation and ATP depletion.

Lipid peroxidation arises when oxidants, like free radicals, interact with lipids that contain carbon–carbon double bonds, especially polyunsaturated fatty acids. Elevated ROS levels can directly damage lipids.^42^ Our results suggest that maternal infection significantly elevates the levels of mitochondrial ROS and polyunsaturated fatty acids within the hearts of offspring mice, creating conditions conducive to lipid peroxidation. Lipid peroxidation, or the reaction of oxygen with unsaturated lipids, generates various oxidative byproducts, including MDA and 4-HNE. Previous research shows that MDA and 4-HNE significantly impact mitochondrial function, including ATP production.^43^^,^^44^ In this study, lipid peroxidation products MDA and 4-HNE increased in the hearts of offspring mice following LPS and Poly(I:C) treatment. A similar elevation was displayed in the *in vitro* hiPSC-CMs with Poly(I:C) challenge, but not in the LPS group. Since ATP reduction was also absent in the LPS-treated group, this suggests that elevated lipid peroxidation products may mediate ATP reduction induced by LPS and Poly(I:C) both *in vivo* and *in vitro*. The differential effects of LPS and Poly(I:C) on lipid peroxidation *in vitro* may be due to their distinct downstream mechanisms. LPS activates immune responses via TLR4, while Poly(I:C) signals through TLR3. TLR4 activation typically occurs via the MyD88-dependent pathway, driving a strong inflammatory response, particularly through cytokines such as TNF-α and IL-6, which amplify immune reactions and oxidative stress. On the other hand, Poly(I:C) activation of TLR3 is not entirely dependent on nuclear factor kappa B (NF-κB)-driven inflammation but relies more on TRIF (TIR (Toll/interleukin-1 receptor) domain-containing adaptor protein inducing interferon beta)-dependent signaling, which leads to antiviral immune responses and interferon production.^45^, ^46^, ^47^ Therefore, in the hiPSC-CM model, the absence of immune cells and the complexity of systemic immune interactions may prevent LPS from fully activating the NF-κB pathway and related cytokines, resulting in a lack of significant lipid peroxidation. Furthermore, the antioxidant Fer-1, which inhibits lipid peroxidation,^48^ rescued ATP levels, and the mitochondrial mt-Ts2 to nuclear DNA ratio decreased by infection, further supporting that lipid peroxidation may mediate mitochondrial damage induced by LPS and Poly(I:C).

### Clinical perspectives

Maternal infections during pregnancy have been linked to adverse fetal cardiac development, yet the underlying mechanisms remain unclear. Our findings suggest that maternal infection may have significant effects on the cardiovascular health of offspring, especially through altered metabolic programming and mitochondrial dysfunction. Future research into therapeutic interventions, such as antioxidants (*e.g.*, NAC or Fer-1), may offer promising avenues for mitigating the impact of maternal infection on cardiac development. These interventions hold promise as potential strategies to prevent or treat fetal cardiac abnormalities associated with maternal infection, opening new clinical avenues for improving maternal and fetal health. However, before these results can be translated into clinical practice, further researches are needed to understand the mechanisms by which maternal infection leads to these developmental changes and whether these effects are reversible or mitigated by early interventions.

### Limitations

This study has some limitations. While we observed certain differences in the impact of LPS and Poly(I:C)-induced maternal infections on the offspring mouse heart, the specific differences and mechanisms were not further explored. Regarding hiPSC-CMs, cells were derived from two healthy donors, and interindividual variability, as well as differences between mice and humans, cannot be excluded. It is also unable to rule out differences in differentiation between hiPSC-CMs and native cardiomyocytes. The main limitation of hiPSC-CMs is their immature phenotype. The immature nature of hiPSC-CMs leads to altered expression profiles of ion channels, sarcomeric proteins, and metabolic enzymes. Furthermore, it is essential to consider that there might be variations in the differentiation process when comparing hiPSC-CMs with native cardiac myocytes. Moreover, although LPS and Poly(I:C) are effective *in vitro* models for initiating cellular inflammatory responses, they do not fully replicate the complex immune environment *in vivo*. These points should be addressed in future studies.

## Conclusions

Our findings indicate that maternal LPS and Poly(I:C) challenges lead to metabolic signaling disruption and mitochondrial dysfunction in offspring heart development, with oxidative stress and lipid peroxidation playing key roles in this process.

## CRediT authorship contribution statement

**Yingrui Li:** Writing – original draft, Visualization, Validation, Resources, Project administration, Methodology, Investigation, Funding acquisition, Formal analysis, Data curation, Conceptualization. **Willfredius Mugishagwe Rutahoile:** Writing – original draft, Visualization, Validation, Formal analysis, Conceptualization. **Binquan Xiong:** Project administration, Methodology, Investigation, Data curation. **Jianlin Du:** Writing – review & editing, Project administration, Investigation, Funding acquisition. **Songbai Deng:** Writing – original draft, Investigation. **Bin Liu:** Investigation, Funding acquisition. **Xiaodong Jing:** Investigation. **Huiping Yang:** Investigation. **Yue Han:** Investigation. **Qiang She:** Writing – review & editing, Supervision, Project administration, Investigation, Funding acquisition, Conceptualization.

## Ethics declaration

All animal experiments adhered strictly to the ARRIVE guidelines and regulations on laboratory animal management by the National Research Council's Guide for the Care and Use of Laboratory Animals, and were approved by the Institutional Animal Care and Use Committee (IACUC) of the Second Affiliated Hospital of Chongqing Medical University (IACUC-SAHCQMU-2024-00013). Title of the approved project is “Mechanistic Study on the Inhibition of Myocardial Development by Infection During Pregnancy” (Date of approval: February 22, 2024).

## Data availability

The expression matrix for mRNA sequencing and the data for non-targeted metabolomics are provided as supplemental materials within the manuscript (Tables S1 and S2). The data that support the findings of the current study are available from the corresponding author upon reasonable request.

## Funding

This study was supported by the 10.13039/501100001809National Natural Science Foundation of China (No. 82270281, 82400365), the Key Project of Technology Innovation and Application Development in Chongqing, China (No. CSTB2023TIAD-KPX0048), 10.13039/501100002858China Postdoctoral Science Foundation (No. 2023MD744155), the Natural Science Foundation of Chongqing Science and Technology Commission (China) (No. CSTB2023NSCQ-BHX0020), Construction of Graduate Tutor Team in Chongqing Medical University (China) (No. cqmudstd202205), Chongqing Elite Teachers and Experts Project (China) (QiangShe [2022]), Postdoctoral Project of Chongqing Natural Science Foundation (China) (No. CSTB2022NSCQ-BHX0626), Senior Medical Talents Program of Chongqing for Young and Middle-aged (China) (JianlinDu [2022]), Future Medicine Youth Innovation Team Development Support Program of Chongqing Medical University (China) (No. W0133), and Kuanren Talents Program of the Second Affiliated Hospital of Chongqing Medical University (China).

## Conflict of interests

The authors declared no competing interests.
