# Supplementary material for: Impact of maternal lipopolysaccharide and polyinosinic-polycytidylic acid-induced infections on offspring cardiac development: Mitochondrial dysfunction and metabolic alterations
Source: Genes Dis. 2025 Oct 8;13(4):101877. doi: 10.1016/j.gendis.2025.101877 (PMC13090308; doi:10.1016/j.gendis.2025.101877)
Supplement: Multimedia component 1 [file mmc1.docx]

***Supplementary Matrial***

**Impact of Maternal Lipopolysaccharide and Polyinosinic-polycytidylic acid Induced Infections on Offspring Cardiac Development: Mitochondrial Dysfunction and Metabolic Alterations**

Yingrui Li^#1^, Willfredius Mugishagwe Rutahoile^#1^, Binquan Xiong^1^, Jianlin Du^1^, Songbai Deng^1^, Bin Liu^1^, Xiaodong Jing^1^, Huiping Yang^1^, Yue Han^1^, Qiang She^*1^

^1^Department of Cardiology, The Second Affiliated Hospital of Chongqing Medical University, Chongqing, China, 400010

#These authors contributed equally: Yingrui Li, Willfredius Mugishagwe Rutahoile

*Correspondence: Qiang She (qshe98@cqmu.edu.cn)

Address for correspondence: Department of Cardiology, The Second Affiliated Hospital of Chongqing Medical University, No. 74, Linjiang Road, Yuzhong District, Chongqing, China, 400010

Tel.: +86 23 63693753

**Materials and methods**

**Animal Model**

C57BL/61 mice were utilized for all animal procedures, which were housed in an individual ventilated cage (IVC)-grade animal facility at the Second Affiliated Hospital of Chongqing Medical University. The temperature in the animal room was maintained at 22–26°C, with relative humidity kept between 40% and 60%. All procedures adhered strictly to the ARRIVE guidelines and regulations on laboratory animal management by the National Research Council's Guide for the Care and Use of Laboratory Animals, and were approved by the Institutional Animal Care and Use Committee (IACUC) of the Second Affiliated Hospital of Chongqing Medical University (IACUC-SAHCQMU-2024-00013). Mice aged 10-12 weeks and weighing 20-30 g were used for the experiments. Mice that died during the experiment or did not meet the inclusion criteria were excluded. The 36 male C57BL/6 mice and 66 female C57BL/6 mice were housed in groups of 1 male and 2 females or 2 males and 3 females, and were placed together at 16:00. The following morning at 08:00, vaginal plugs were checked, and the day of the vaginal plug was recorded as embryonic day E0.5. To establish the maternal infection model, LPS (Sigma#L2630) and Poly(I:C) (Sigma# P1530) were administered via intraperitoneal injection to pregnant mice at E16.5, with doses based on previous studies ^[14]^. The hearts of newborn offspring mice [within 12 hours of birth, postnatal day (P)0] and 4-week-old offspring mice were used for further studies. No animals were excluded during the experiments. In order to minimize potential confounders, all animals were housed in a manner that minimized environmental factors that could affect the outcomes. All animals were cervical dislocation under inhaled isoflurane anesthesia after the experiments.

**Human Induced Pluripotent Stem Cells Culture and Cardiomyocyte Differentiation**

Two human induced pluripotent stem cell (hiPSC) lines, F1 and B1, were generated from two healthy donors: 29-year-old female skin fibroblast-derived cells (F1) and 28-year-old female blood-derived cells (B1). These cells were purchased from Beijing Cellapy Biotechnology (China). Culture plates and dishes were coated with Matrigel (Corning). According to manufacturer protocols, hiPSCs were resuscitated in PSCeasy thawing medium (Cellapy Biotechnology, #CA1003100). The hiPSCs were maintained in PGMI pluripotent stem cell culture medium (Cellapy Biotechnology, #CA1007500). After 4–5 days of culture, when the cells reached 85–95% confluence, they were passaged with EDTA onto new plates. To differentiate the hiPSCs into cardiomyocytes, *in vitro* cardiomyocyte differentiation was performed using a cardiomyocyte differentiation kit (Beijing Cellapy Biotechnology, #CA2004500), following the user’s instructions. Differentiation was considered successful when cells began to beat between day 8 and 10. From days 12–15 of differentiation, cardiomyocyte purification medium (Beijing Cellapy Biotechnology, #CA2005100) was used to purify the differentiated cardiomyocytes. The differentiation of each hiPSC cell line into hiPSC-derived cardiomyocytes (hiPSC-CMs) was performed every 4–6 days, and the data from different differentiations were combined for statistical analyses. Both cell lines (F1 and B1) were differentiated in parallel using the same protocol and conditions. LPS (Sigma, #L2630) or Poly(I:C) (Sigma, #P1530) was applied during hiPSC-CM differentiation (days 16–20), and cells at day 20 were used for subsequent experiments to observe the effects on cardiomyocyte differentiation.

**mRNA Sequencing and Bioinformatic Analysis**

Hearts from the newborn offspring mice in each group were collected, and RNA was extracted using Trizol for subsequent sequencing. RNA sequencing was performed by Sangon Biotech (Shanghai, China). Briefly, 1 μg of RNA from each sample was used as input to prepare the RNA samples. cDNA libraries were constructed using the Hieff NGS™ MaxUp Dual-mode mRNA Library Prep Kit for Illumina® (YEASEN, #12301ES96) according to the manufacturer's instructions, and sequencing was conducted on a DNBseq-T7 sequencer (PE150). Raw sequencing data were assessed for quality using FastQC (version 0.11.2), and quality trimming was performed with Trimmomatic (version 0.36) to obtain relatively accurate and usable data. The effective data from each sample were aligned to the reference genome using HISAT2 (version 2.0), and mapping statistics were generated. Redundant sequence analysis and fragment insertion distribution were conducted using RSeQC (version 2.6.1). Uniformity distribution checks and genomic structure distribution were performed using Qualimap (version 2.2.1). Gene coverage and the distribution of sequencing reads across chromosomes were analyzed using BEDTools (version 2.26.0). Gene expression levels were evaluated using StringTie (version 1.3.3b) based on known gene models. The TPM (Transcripts Per Million) metric was used to eliminate the effects of gene length and sequencing discrepancies, enabling direct comparison of gene expression between samples (**Supplemental Table S1**). Differential gene expression was determined via DESeq2 (version 1.12.4) with significance thresholds set at |FoldChange| ≥1 and adjusted q-value ≤ 0.05.

Gene expression differences were visualized using violin plots, heatmaps, and volcano plots. The violin plots of gene expression were generated in GraphPad (version 8.0.2), while heatmaps and volcano plots of the DEGs were created using the R platform. The DEGs were further analyzed using GO and KEGG enrichment analyses with the clusterProfiler package. Functional enrichment was considered significant when the adjusted P-value (Q-value) < 0.05. The GO annotation included biological processes (BP), cellular components (CC), and molecular functions (MF). The categories of KEGG pathways included Cellular Processes, Environmental Information Processing, Genetic Information Processing, Metabolism, Organismal Systems, Human Diseases, and Drug Development (<https://www.genome.jp/kegg/pathway.html>).

**Nontargeted Metabolomics**

After perfusion with pre-cooled PBS buffer, the hearts of the newborn offspring mice from each group were quickly excised, frozen in liquid nitrogen, and stored at -80°C for further analysis. Gas chromatography-mass spectrometry (GC-MS) and liquid chromatography-mass spectrometry (LC-MS) nontargeted metabolomics were conducted by Oebiotech (Shanghai, China).

**Sample Preparation**. 20 mg of each sample were placed into 1.5 mL centrifuge tubes. To each tube, 20 μL of internal standard (2-chloro-l-phenylalanine in methanol, 0.3 mg/mL) and 400 μL of methanol-water (v: v=4:1) were added. The samples were placed in a -40°C freezer for 2 minutes, then ground using a grinder (60 Hz, 2 minutes). The samples were subjected to ultrasonic extraction in an ice-water bath for 10 minutes and left at -40°C overnight. On the following day, the samples were centrifuged at 12,000 rpm for 10 minutes at 4°C, and 150 μL of the supernatant was collected. After filtration through a 0.22 μm organic-phase syringe filter, the samples were transferred into LC vials and stored at -80°C for LC-MS analysis.

In parallel, 150 μL of the supernatant was placed into glass derivatization vials, and the samples were concentrated using a centrifugal concentrator. Then, 80 μL of methoxyamine hydrochloride pyridine solution (15 mg/mL) was added, and the samples were shaken at 37°C for 60 minutes. After this, 50 μL of bis(trimethylsilyl)fluoroacetamide (BSTFA) derivatization reagent and 20 μL of n-hexane were added, along with 10 internal standards (C8/C9/C10/C12/C14/C16/C18/C20/C22/C24, all in chloroform solution, 10 μL each). The samples were reacted at 70°C for 60 minutes and then kept at room temperature for 30 minutes before undergoing GC-MS metabolomics analysis. The quality control (QC) sample was prepared by pooling aliquots from all samples.

**LC-MS Analysis.** Samples were analyzed on an ACQUITY UPLC I-Class system (Waters Corporation, Milford, USA) coupled with a Q-Exactive quadrupole-Orbitrap mass spectrometer (Thermo Fisher Scientific, Waltham, MA, USA) with a heated electrospray ionization (ESI) source. An ACQUITY UPLC HSS T3 column (1.8 μm, 2.1 × 100 mm) was used in both positive and negative ion modes. The binary gradient elution system consisted of (A) water (containing 0.1% formic acid, v/v) and (B) acetonitrile (containing 0.1% formic acid, v/v), with the following gradient: 5–20% B from 0–2 min, 20–60% B from 2–4 min, 60–100% B from 4–11 min, held at 100% B for 2 min, then 100% to 5% B from 13 to 13.5 min, and held at 5% B from 13.5 to 14.5 min. The flow rate was 0.35 mL/min, and the column temperature was set to 45°C. The injection volume was 3 μL. The mass range was m/z 70–1050. The resolution was set at 70,000 for full MS scans and 17,500 for MS/MS scans. The collision energy was set to 10, 20, and 40 eV. The mass spectrometer settings were as follows: spray voltage, 3800 V (+) and 3000 V (−); sheath gas flow rate, 35 arbitrary units; auxiliary gas flow rate, 8 arbitrary units; capillary temperature, 320°C.

Progenesis QI (Waters Corporation) processed LC-MS raw data through baseline adjustment, peak annotation, retention time calibration, alignment, and normalization. Precursor tolerance was set to 5 ppm, fragment tolerance to 10 ppm, and retention time tolerance to 0.02 min. Compound identification was based on precise m/z ratios, secondary fragments, and isotopic distributions using the Human Metabolome Database (HMDB), Lipidmaps (V2.3), METLIN, and a self-constructed database (LuMet-Animal 3.0) for qualitative analysis. Data preprocessing involved handling missing values, replacing zero values, applying scoring filters, and merging datasets. Peaks with >50% missing values within a group were excluded, and remaining zeros were replaced with half the minimum ion intensity of all samples. Compound scores were used for filtering, with a cutoff of 36 (out of 80) for valid identifications. The final matrix of data was merged with the GC-MS data, providing a comprehensive dataset for subsequent analysis.

**GC-MS Analysis.** The derivatized samples were analyzed using an Agilent 8890-5977B gas chromatography system coupled to an Agilent 5977A MSD system (Agilent Technologies Inc., CA, USA). A DB-5MS fused-silica capillary column (30 m × 0.25 mm × 0.25 μm, Agilent J & W Scientific) was used to separate the derivatives. Helium (>99.999%) was used as the carrier gas at a constant flow rate of 1 mL/min. The injector temperature was set to 260°C, and the injection volume was 1 μL (splitless mode). The oven temperature program was as follows: 60°C for 2 minutes, ramped to 125°C at 8°C/min, to 210°C at 5°C/min, to 270°C at 10°C/min, to 305°C at 20°C/min, and held at 305°C for 5 minutes. The temperature of the MS quadrupole and ion source (electron impact) was set to 150°C and 230°C, respectively. The collision energy was 70 eV. Data were collected in full-scan mode (m/z 50-500), with a solvent delay time of 5 minutes. QC samples were injected at regular intervals (every 3 samples) to assess repeatability.

The raw GC-MS data were converted from the .D format to .abf format using AnalysisBaseFileConverter software and then imported into MD-DIAL software for data processing. Metabolites were annotated using the LUG database (Untargeted database for GC-MS from Lumingbio). After aligning the data, the “raw data array” was generated, containing sample information, peak names (or retention times and m/z), and peak intensities. In the data quality control process, internal standard peaks and any known false positives (such as noise, column bleed, and derivatization reagent peaks) were removed. Peaks with >50% missing values were excluded, and remaining missing values were replaced by half the minimum value. Data were normalized using an internal standard-based normalization algorithm, and the data matrix was refined. Based on compound qualitative results, a scoring threshold of 70 (out of 100) was applied to retain accurate identifications.

The data matrix was then combined with LC-MS/MS data (both positive and negative ions) to create a final dataset for subsequent analysis (**Supplemental Table S2**). Principal Component Analysis (PCA) and Orthogonal Partial Least Squares-Discriminant Analysis (OPLS-DA) were conducted to visualize metabolic differences across experimental groups, following mean centering (Ctr) and Pareto scaling (Par). The Hotelling's T2 region in the score plots defined the 95% confidence interval. Variables with VIP > 1 were considered relevant for group discrimination. Cross-validation (7 rounds) was performed to guard against overfitting. Two-tailed Student’s T-test was used to verify the significance of metabolite differences, and differential metabolites with VIP > 1.0 and p < 0.05 were selected. Heatmaps and volcano plots of the differential metabolites were drawn in the R platform, and KEGG pathway enrichment analysis was performed for the differential metabolites.

**Transmission Electron Microscopy**

Offspring mouse hearts were isolated with minimal mechanical damage, such as pulling, crushing, or squeezing. Sampling was completed within 1-3 minutes. The tissue was fixed in electron microscopy fixative (biossci#BP0130) in an EP tube. The samples were then fixed with 1% osmium tetroxide in 0.1M phosphate buffer (PB, pH 7.4) at room temperature for 2 hours, protected from light. The tissue was washed three times with 0.1M phosphate buffer (pH 7.4), 15 minutes each time. Dehydration was performed using ascending concentrations of ethanol: 30%, 50%, 70%, 80%, 95%, 100%, and 100% ethanol, each for 10 minutes. The tissue was then rinsed twice with 100% acetone, 10 minutes each time. After dehydration, the tissue was infiltrated and embedded with acetone and embedding agent 812 (SPI#02660-AB). The embedded samples were sectioned to 70 nm thickness (Leica#Leica UC7) and observed under a transmission electron microscope (HITACHI#HT7700).

**Immunofluorescence**

Newborn offspring mouse hearts were isolated and fixed in 4% paraformaldehyde overnight at 4°C. The samples were then dehydrated with ethanol, cleared with Histoclear, and embedded in paraffin. Paraffin-embedded sections were dewaxed, rehydrated, treated with blocking buffer (10% serum in TBST) for 30 minutes, incubated with the primary antibody overnight at 4°C, and incubated with an Alexa-conjugated secondary antibody (Thermo Fisher) at room temperature for 45 minutes. Slides were then washed thoroughly with TBST and briefly stained with DAPI (Solarbio #C0060) to visualize the nuclei. If the primary antibody was homologous, TSA amplification was used for staining.

For hiPSCs and hiPSC-CMs, cells were fixed with 4% paraformaldehyde at room temperature for 15 minutes, permeabilized with 0.25% Triton X-100 for 5 minutes, and blocked with 5% BSA at room temperature for 1 hour. The primary antibody was incubated overnight at 4°C. The next day, cells were washed with PBS three times for 5 minutes each, then incubated with the secondary antibody at 37°C in the dark for 45 minutes. The cells were then stained with 4',6-diamidino-2-phenylindole (DAPI) at room temperature in the dark for 5 minutes. The primary antibodies used for immunostaining was purchased from Abcam (TNNT2, Proteintech#15513-1-AP; ACTN, Proteintech#11313-2-AP; KI67，Proteintech#27309-1-AP; OCT4，Proteintech#11263-1-AP; NANOG1，Proteintech#14295-1-AP; 4-HNE，Bioss#bs-6313R). All immunostained samples were observed using a fluorescence confocal microscope (Olympus).

**Flow Cytometry**

hiPSC-CMs were digested with cardiac myocyte digestion solution (Beijing Cellapy Biotechnology #CA2011100 and #CA2012100) and fixed overnight at -20°C with 70% cold ethanol. On the following day, cells were grouped to ensure a concentration of 5x10^5^ to 1x10^6^ cells per group. Cells were first stained with AlexaFluor 647-TNNT2 (BD Biosciences #565744) in Permeabilization Buffer (Thermo #00-8333-56) at room temperature for 60 minutes. The samples were then analyzed using the CytoFLEX Flow Cytometer (Beckman Coulter). The geometric mean fluorescence intensity (MFI) of TNNT2 was quantified, and data were analyzed using CytExpert 2.4.

**Western Blot**

Total protein from tissues and cells was extracted using RIPA buffer (Beyotime #P0013B) with Protease Inhibitor Cocktail (Beyotime #P1005). The total protein concentration was measured using a BCA Protein Assay Kit (Beyotime #P0012S). For the protein measurement, 15 µg of total protein per sample was used. SDS-PAGE was performed to separate the proteins based on their size. Western blotting was carried out using the Bio-Rad Mini-PROTEAN® electrophoresis system (Bio-Rad, Inc. #1658033) according to the manufacturer's instructions, with PVDF membranes used for blotting. The primary antibody was incubated overnight, followed by incubation with a conjugated secondary antibody for 1 hour the next day. Non-specific binding sites were blocked with milk powder. Primary antibodies used included TNNT2 (Proteintech#15513-1-AP), TNNI3 (Proteintech#21652-1-AP), and β-tubulin (Proteintech#10094-1-AP), and the secondary antibody used was Goat Anti-Rabbit IgG H&L (Abcam #ab205718). After antibody incubation, chemiluminescent detection was performed using a detection reagent (Thermo Fisher Scientific #33209), and the membranes were exposed using the Bio-Rad Chemidoc Imaging System.

**Quantitative Reverse Transcription Polymerase Chain Reaction**

Total RNA from the hearts of newborn offspring mice and cells was extracted using the Trizol method. For cDNA synthesis, 1 µg of RNA was reverse transcribed using the PrimeScript RT Reagent Kit with gDNA Eraser (Takara) according to the manufacturer's instructions. qPCR was performed on the CFX Connect Real-Time PCR Detection System (Bio-Rad) using the TB Green Premix Ex Taq™ II (Tli RNaseH Plus) (Takara). TATA-binding protein (TBP) was used as the housekeeping gene. The primer pairs used are listed in **Supplemental Table S3**.

**Mitochondrial DNA (MtDNA) Analysis**

Total DNA was extracted from the hearts of newborn offspring mice and hiPSC-CMs using a DNA extraction kit (Servicebio #G3633), following the manufacturer's instructions. qPCR analysis was performed on the CFX Connect Real-Time PCR Detection System (Bio-Rad) using primers specific for mitochondrial DNA. The primer pairs are listed in **Supplemental Table S1**.

**ATP Content Measurement**

ATP content in the hearts of newborn offspring mice and cells was measured using the CheKine™ ATP Content Assay Kit (Abbkine #KTB1016), according to the manufacturer's instructions. Luciferase activity was measured using a Spectrophotometer (Thermo Fisher). The raw values were normalized to protein concentration.

**MitoTracker and MitoSOX Red**

To assess mitochondrial bioactivity and membrane potential, Mito-Tracker Red CMXRos (Beyotime #C1049B) was used. To detect mitochondrial superoxide, MitoSOX Red (MedChemExpress #HY-D1055) was employed. For the hearts of newborn offspring mice, tissues were extracted and embedded in optimal cutting temperature (OCT). The OCT-embedded sections were then treated with Mito-Tracker Red CMXRos or MitoSOX Red for 30 minutes. For hiPSC-CMs, cells were treated with Mito-Tracker Red CMXRos or MitoSOX Red for 30 minutes on day 20 of differentiation. Nuclei were stained with DAPI. Samples were observed using a fluorescence confocal microscope (Olympus).

**Oxidized Glutathione (GSSG) and** **Total Glutathione (T-GSH)**

GSSG and T-GSH levels in the hearts of newborn offspring mice and cells were measured using the T-GSH/GSSG Colorimetric Assay Kit (Elabscience #E-BC-K097-M), following the manufacturer's instructions. Luciferase activity was measured using a Spectrophotometer (Thermo Fisher). The raw values from tissues were normalized to tissue weight, while the raw values from cells were normalized to cell concentration.

**Malondialdehyde (MDA) Content**

MDA content in the hearts of newborn offspring mice and cells was measured using the Lipid Peroxidation MDA Assay Kit (Beyotime #S0131), according to the manufacturer's instructions. Luciferase activity was measured using a Spectrophotometer (Thermo Fisher). The raw values were normalized to protein concentration.

**Statistical Analysis**

Statistical analysis of the data was performed using SigmaPlot 14.0 (Systat GmbH, Germany). Mean values and standard errors were first calculated. The Kolmogorov-Smirnov test was applied to assess the normality of the data distribution. For comparisons between two independent groups, an unpaired Student's t-test was used. For comparisons involving more than two groups, one-way analysis of variance (ANOVA) followed by a Holm-Sidak post-test for multiple comparisons was conducted. P-values < 0.05 were considered statistically significant.

**Figure S1**


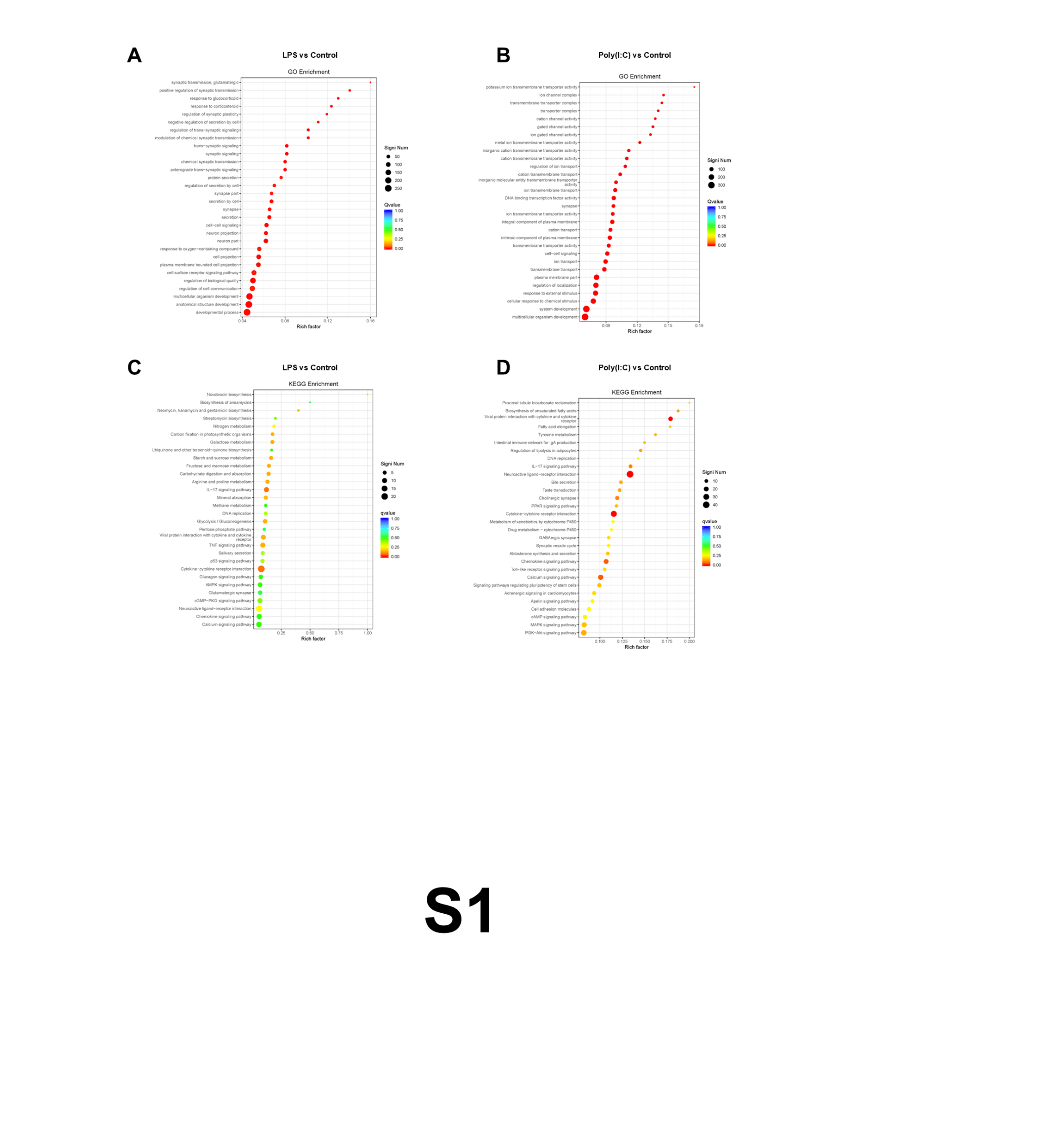


**Figure S2**


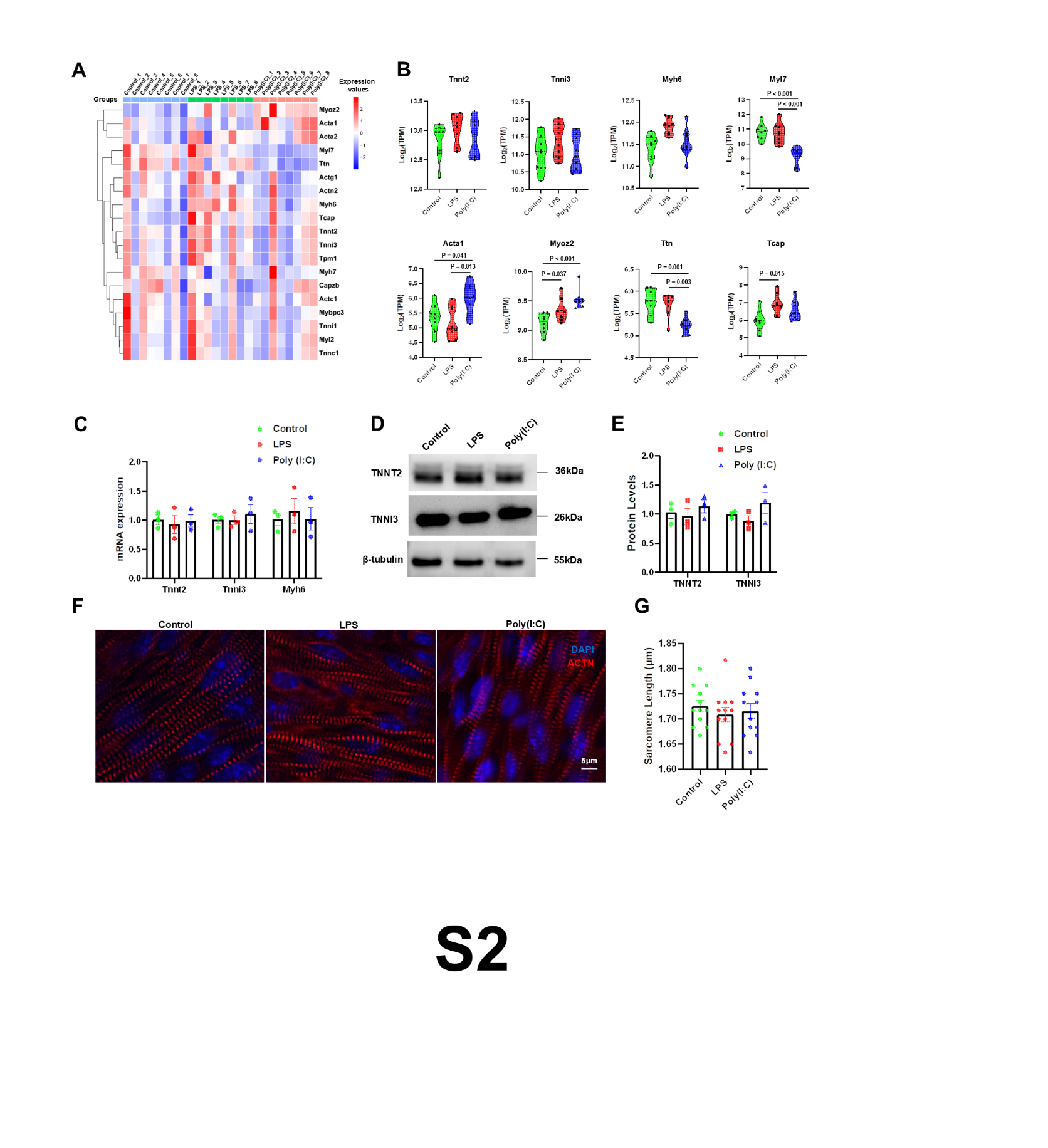


**Figure S3**


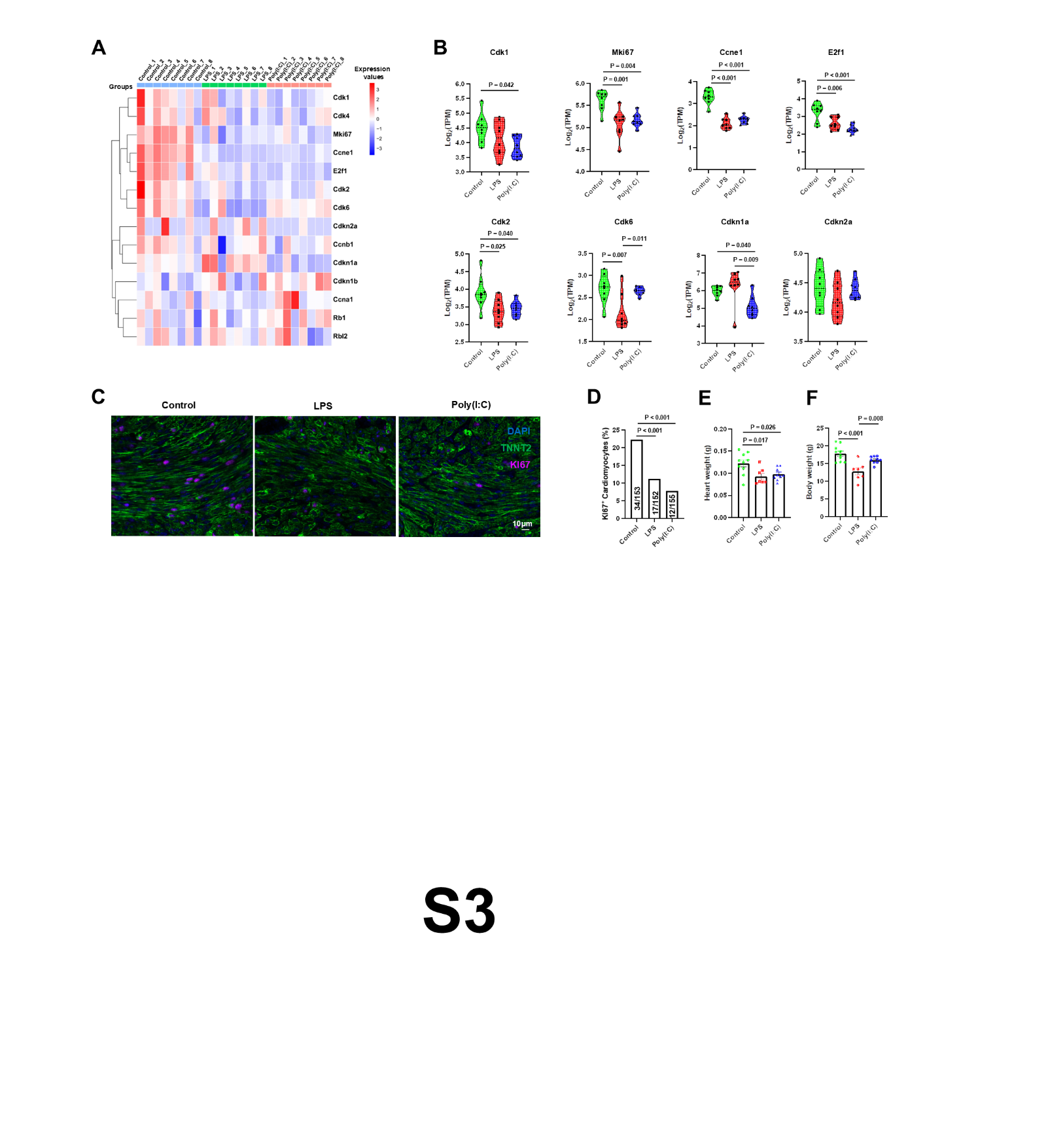


**Figure S4**


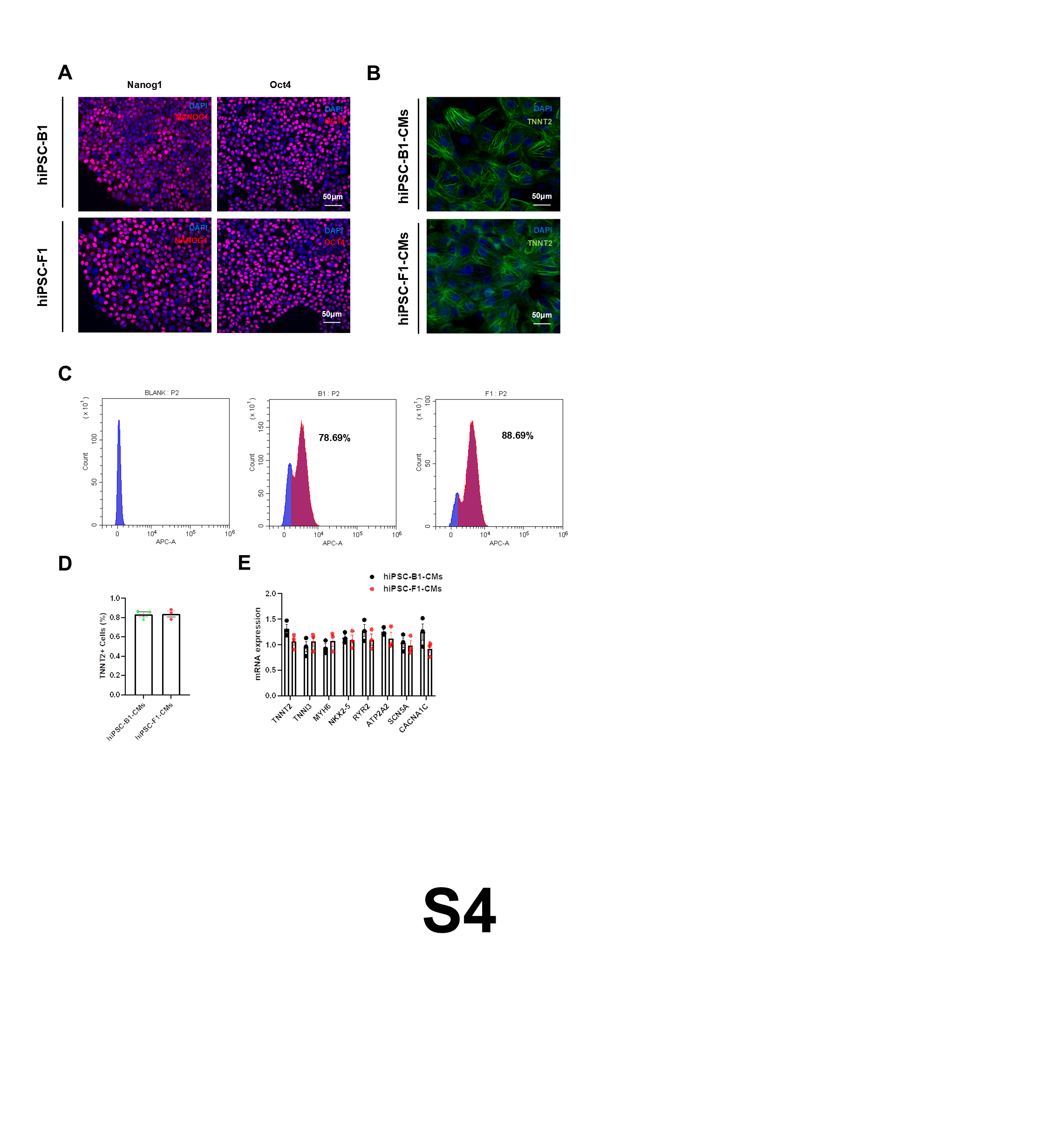


**Figure S5**


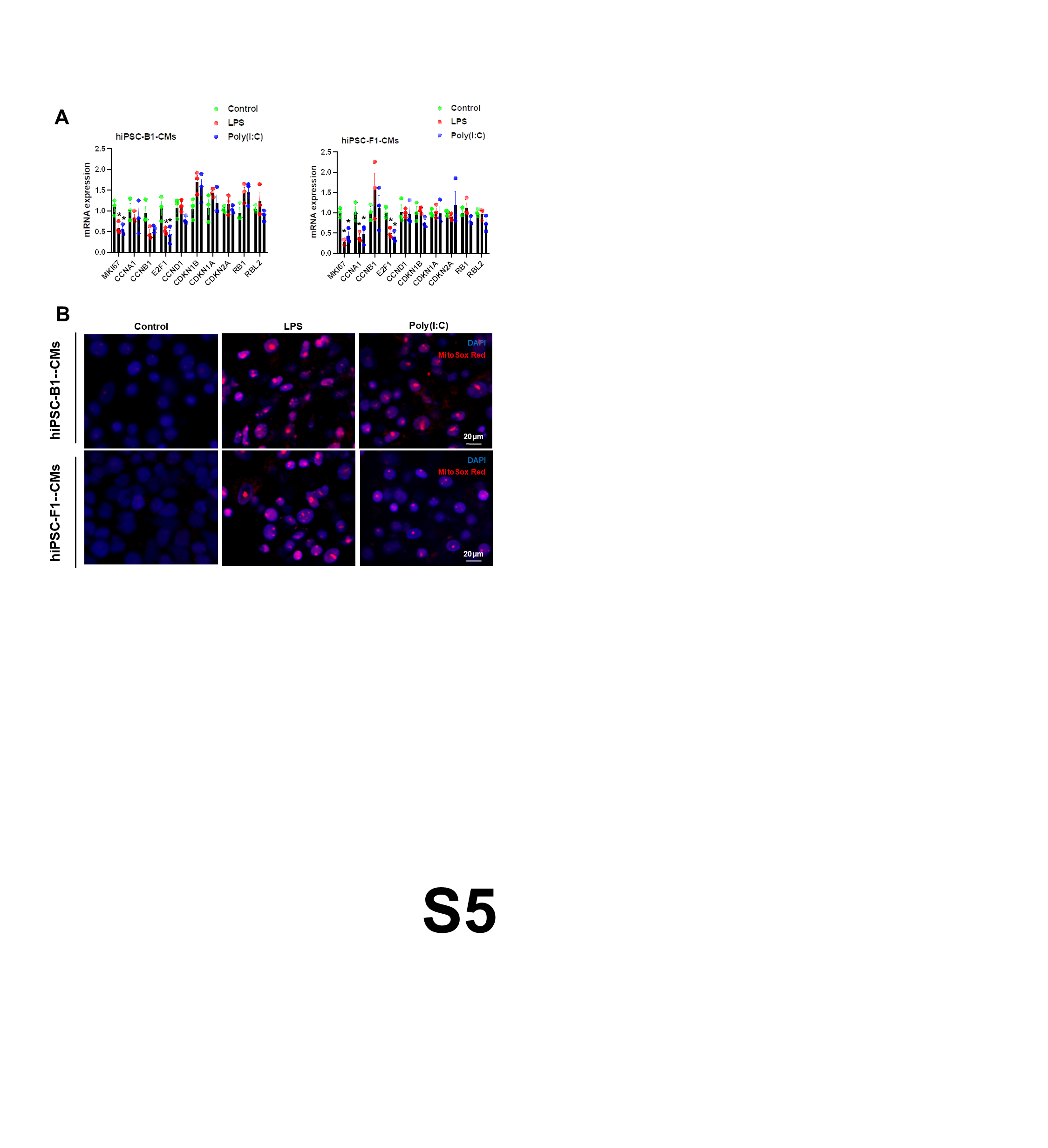


**Figure S6**


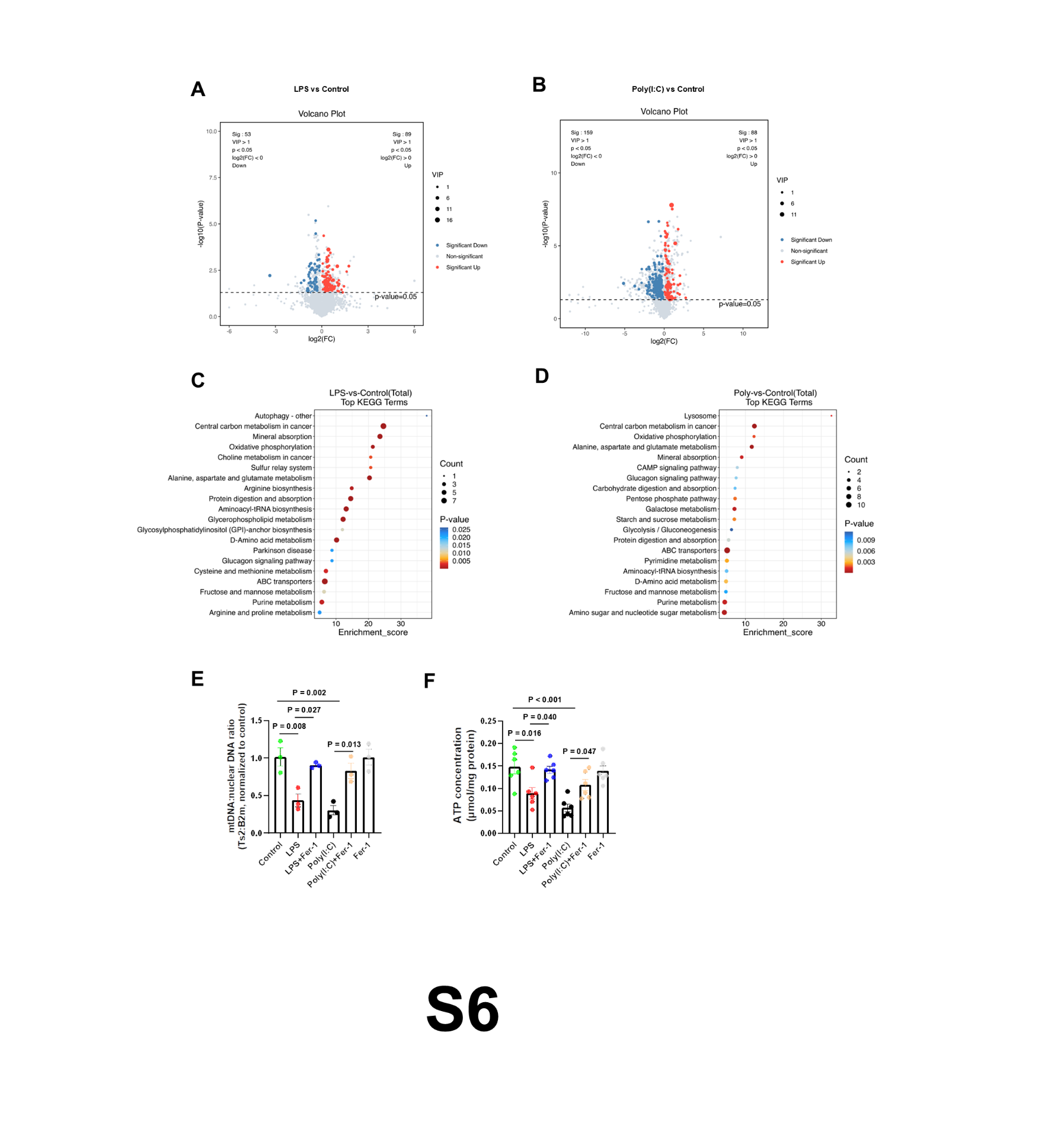


**Figure Legends**

**Figure S1. Enrichment analysis of differentially expressed genes in the hearts of offspring mice following maternal infection.** Pregnant mice were intraperitoneally injected with LPS (300ug/kg) or Poly(I:C) (20mg/kg) at E16.5. The hearts of the newborn offspring mice were extracted within 12 hours of birth [postnatal day (P)0] for mRNA sequencing and bioinformatic analysis. (A) GO enrichment analysis of differentially expressed genes between Control and LPS. (B) GO enrichment analysis of differentially expressed genes between Control and Poly(I:C). (C) KEGG enrichment analysis of differentially expressed genes between Control and LPS. (D) KEGG enrichment analysis of differentially expressed genes between Control and Poly(I:C).

**Figure S2. Effect of maternal infection on sarcomere maturation in the hearts of offspring mice.** Pregnant mice were intraperitoneally injected with LPS (300ug/kg) or Poly(I:C) (20mg/kg) at E16.5. The hearts of the newborn offspring mice were extracted within 12 hours of birth [postnatal day (P)0] for mRNA sequencing and bioinformatic analysis. (A) Heat map of sarcomere-related genes among Control, LPS, and Poly(I:C). (B) Violin plots of the expression of sarcomere-related genes among Control, LPS, and Poly(I:C). (C) The fold change in mRNA expression levels of cardiomyocyte sarcomere markers in Control, LPS, and Poly(I:C). (D-E) Bands and mean values of Western blots showing TNNT2 and TNNI3 levels in tissues with or without LPS or Poly(I:C) treatment. (F) Sarcomeric structure in Control, LPS, and Poly(I:C) groups (bar = 5μm). (G) Quantification of sarcomeric length. One-way analysis of variance (ANOVA) followed by Holm-Sidak post-test for multiple comparisons was used to compare more than two groups. The data are presented as mean ± standard error.

**Figure S3. Effect of maternal infection on cardiomyocyte proliferation in the hearts of offspring mice.** Pregnant mice were intraperitoneally injected with LPS (300ug/kg) or Poly(I:C) (20mg/kg) at E16.5. The hearts of the newborn offspring mice were extracted within 12 hours of birth [postnatal day (P)0] for mRNA sequencing and bioinformatic analysis. (A) Heat map of proliferation-related genes among Control, LPS, and Poly(I:C). (B) Violin plots of the expression of proliferation-related genes among Control, LPS, and Poly(I:C). (C) Representative images of heart tissue stained with Ki67/TNNT2/DAPI in each group (bar = 10μm). (D) The percentage of total DAPI/TNNT+ cardiomyocytes that are Ki67+ or Ki67- in Control, LPS, and Poly(I:C) groups. One-way analysis of variance (ANOVA) followed by Holm-Sidak post-test for multiple comparisons was used for comparing more than two groups. The Fisher test was applied for comparing categorical variables. The data are presented as mean ± standard error.

**Figure S4. Expression of cardiac-specific markers and pluripotency markers.** (A) Immunofluorescence analysis of pluripotency markers in hiPSC-B1 and hiPSC-F1 cell lines. (B) Immunofluorescence analysis of the cardiac-specific marker TNNT2 in hiPSC-B1-CMs and hiPSC-F1-CMs cell lines. (C) Flow cytometry analysis for the cardiac-specific marker TNNT2. (D) Percentage of TNNT2+ cardiomyocytes in hiPSC-B1-CMs and hiPSC-F1-CMs. (E) The fold change in mRNA expression levels of cardiomyocyte markers in hiPSC-B1-CMs and hiPSC-F1-CMs cell lines. One-way analysis of variance (ANOVA) followed by Holm-Sidak post-test for multiple comparisons was used for comparing more than two groups. The unpaired Student's t-test was used to compare two independent groups. The data are presented as mean ± standard error.

**Figure S5. Effects of LPS and Poly(I:C) challenge on proliferation markers and mtROS during cardiomyocyte differentiation.** From day 16 to day 20 of hiPSC differentiation into cardiomyocytes, cells were treated with LPS (5ug/ml) and Poly(I:C) (10ug/ml). On day 20 of differentiation, the cells were used for experiments. (A) The fold change in mRNA expression levels of proliferation-related genes in hiPSC-B1-CMs and hiPSC-F1-CMs cell lines in each group. (B) Representative images of hiPSC-B1-CMs and hiPSC-F1-CMs stained with MitoSox Red/DAPI in each group (bar = 20 μm). *P<0.05 by one-way analysis of variance (ANOVA) followed by Holm-Sidak post-test for multiple comparisons. The data are presented as mean ± standard error.

**Figure S6. Differential metabolites and enrichment analysis of the offspring mouse heart in response to maternal infection.** Pregnant mice were intraperitoneally injected with LPS (300ug/kg), Poly(I:C) (20mg/kg) or Fer-1 (5mg/kg) at E16.5. The hearts of the newborn offspring mice were extracted within 12 hours of birth [postnatal day (P)0] for mRNA sequencing and bioinformatic analysis. (A) Volcano plot of differential metabolites in LPS vs Control. (B) Volcano plot of differential metabolites in Poly(I:C) vs Control. (C) KEGG enrichment analysis of differential metabolites between Control and LPS. (D) KEGG enrichment analysis of differential metabolites between Control and Poly(I:C). (E) Ts2 to nuclear B2M DNA ratio in offspring mouse heart tissue in each group. (F) ATP contents in offspring mouse heart tissue in each group. One-way analysis of variance (ANOVA) followed by Holm-Sidak post-test for multiple comparisons was used to compare more than two groups. The data are presented as mean ± standard error.
